# Diversity, phenology and distribution of *Termitomyces* species in Côte d’Ivoire

**DOI:** 10.1080/21501203.2018.1500498

**Published:** 2018-07-30

**Authors:** N’golo Abdoulaye Koné, Bakary Soro, Linda Patricia Louyounan Vanié-Léabo, Souleymane Konaté, Adama Bakayoko, Daouda Koné

**Affiliations:** a Université Nangui Abrogoua, UFR des Natural Sciences (UFR-SN), Unité de Recherche en ecology et Biodiversité (UREB), Station de Recherche en Ecologie du Parc National de la Comoé, Abidjan, Côte d’Ivoire; b WASCAL Graduate Study Program (GSP) Climate Change and Biodiversity, University Félix Houphouët-Boigny, Abidjan, Côte d’Ivoire; c Université Nangui Abrogoua, UFR des Sciences de la Nature, Abidjan, Côte d’Ivoire

**Keywords:** *Termitomyces*, fungus-growing termites, diversity, phenology, distribution, Côte d’Ivoire

## Abstract

The mutualistic symbiosis between termites of the Macrotermitinae subfamily (Isoptera: Termitidae) and fungi of the genus *Termitomyces* (Basidiomycota: Lyophyllaceae) is of great ecological and socio-economic importance. Seasonal fruit bodies of the symbiotic fungi are regularly collected and sold in Côte d’Ivoire. However, there are very few studies on their diversity, phenology, distribution and especially the socio-economic scope of the fruit bodies of these fungi at a national scale. This study aims at (i) assessing the diversity of *Termitomyces* fruit bodies in Côte d’Ivoire and (ii) mapping their fructification areas through a determination of their spatiotemporal distribution according to a climatic and phytogeographic gradients. Using ethnomycological surveys all over the Ivorian territory, information was collected from rural populations on the fructification of *Termitomyces* and their socio-economic importance. Based on these surveys, sampling efforts of these fungi were properly structured and oriented. The results revealed a diversity of 16 species of *Termitomyces*, including 9 species new to Côte d’Ivoire and 2 probably new to science. Five species were found in the forest zone, nine in theGuinean savannah zone and four in the Sudano-Guinean zone. *Termitomyces*’s fructifications were observed throughout the year, with specific period for each species. All listed species are regularly consumed by populations. However, only *Termitomyces letestui* (Pat.) R. Heim and *Termitomyces schimperi* (Pat.) R. Heim are marketed on a relatively large scale.

## Introduction

The Macrotermitinae (Isoptera: Termitidae) subfamily, the so-called fungus-growing termites, is the largest group of termites because of its abundance but mainly because of its ecological role in the structure, dynamics and functioning of tropical ecosystems. They developed a sophisticated mutualistic symbiosis with a kind of fungi (*Termitomyces* spp., Basidiomycotina), which they cultivate on combs constructed from faecal material within their nests (Johnson et al. 1981; Wood and Thomas 1989). This symbiosis firstly occurred with no reversions to free-living states (Aanen et al. 2002) at least 31 million years ago (Nobre et al. 2011) and is obligate for both partners: the termites provide a constant, highly regulated growth environment for their fungal symbionts, while the fungi provide food for the termites. Fungi are cultivated on combs constructed from faecal material within termites’ nests (Wood and Thomas 1989). Entering the symbiosis has allowed the fungi to overcome highly unfavourable ecological conditions, and the termites to exploit complex plant substrates. As in other symbioses, complex patterns of interactions among groups of multiple species are observed.

*Termitomyces* Heim (1942) is a genus of mushrooms whose seasonal edible fructifications are found exclusively in certain regions of Africa and Asia. Singer (1986) also reported them in the tropical countries of the South Pacific. According to Kirk et al. (2001), about 40 species of those mushrooms have been described to date. Within Côte d’Ivoire, the geographical patterns of fungus-growing termites and their symbionts have not been studied systematically. *Termitomyces*’ fruit bodies are exclusively found in the centre of this country (Koné et al. 2011; Koné 2013; Yorou et al. 2014) and subject to massive and uncontrolled harvesting by the rural populations during their respective fructification periods (Koné et al. 2013). Woodland habitats of this part of the country have been identified as the main fructification ecosystems of these organisms, with specific fructification period for each species (Koné et al. 2011).

**Figure 1.**
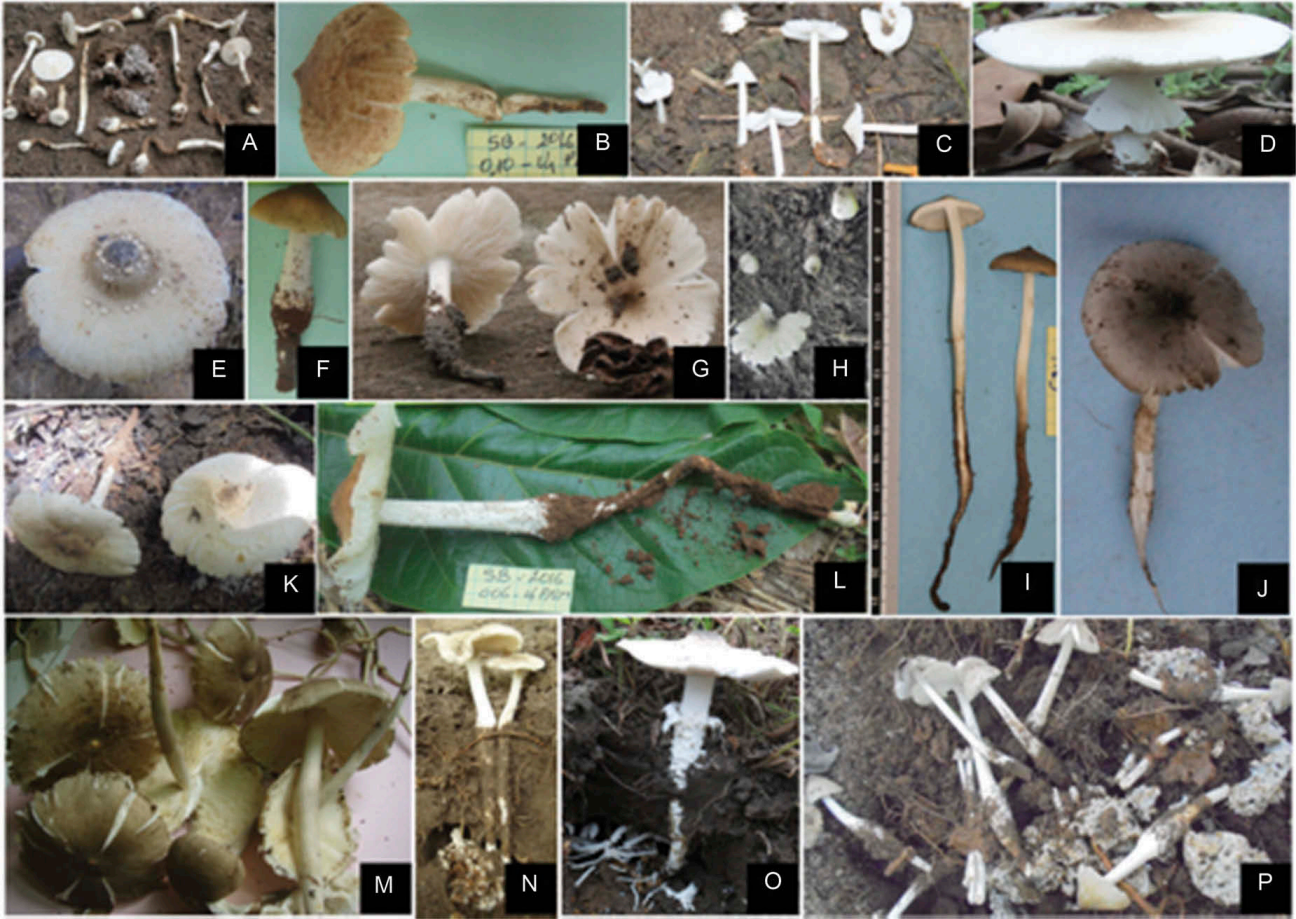
*Termitomyces’* species of Côte d’Ivoire. A = *Termitomyces fuliginosus* R. Heim, B = *Termitomyces* sp6, C = *Termitomyces microcarpus* (Berk. & Broom) Heim, D = *Termitomyces letestui* (Pat.) R. Heim, E = *Termitomyces* sp1, F = *Termitomyces* sp4, G = *Termitomyces* cf. *aurantiacus*, H = *Termitomyces* sp2, I = *Termitomyces* cf. *clypeatus* R. Heim, J = *Termitomyces* sp7, K = *Termitomyces* sp5, L = *Termitomyces* sp3, M = *Termitomyces eurhizus* (Berk.) R. Heim, N = *Termitomyces medius* R. Heim & Grassé, O = *Termitomyces schimperi* (Pat.) R. Heim, P = *Termitomyces striatus* (Beeli) Heim.

Several authors (Tiébré 2001; Koné et al. 2011; Kouassi 2012; Koné 2013; Yorou et al. 2013) identified seven species of *Termitomyces* in this country. However, molecular and phylogenetic studies (Nobré et al. 2011; Koné 2013) estimated that the diversity of *Termitomyces* in Côte d’Ivoire should be more than 20 species. Moreover, it seems that seasonal fructifications of these fungi can be observed in several phytogeographical zones of Côte d’Ivoire. Yet their diversity and especially their spatio-temporal distribution remain hardly documented (Figure 1).

This study aimed at (i) assessing the diversity of *Termitomyces* fruit bodies in Côte d’Ivoire and (ii) mapping their fructification areas through a determination of their spatial and temporal distribution according to their climatic and phytogeographic gradients. In order to achieve these objectives, socio-economic surveys were carried out throughout the Ivorian territory according to the climatic and phytogeographic gradient for collecting information, from rural populations. These ethnomycological surveys were followed by different field surveys in various habitat types of each phytogeographical zone over five consecutive years.

## Materials and methods

### Presentation of the area and study sites

Côte d’Ivoire is characterised by a climate which is generally warm and humid, ranging from equatorial in the southern coasts to tropical in the middle and semi-arid in the far north. Thus, there is a shift from a four-season regime in the South to a two-season regime in the North. The soil types and the topography are obvious factors in vegetation diversification. The vegetation is subdivided into two main Guinean and Sudanese domains respectively composed of two and four sectors with various vegetation types hosting a high biodiversity (Konaté and Linsenmair 2010). In this study, three major phytogeographical zones were considered; namely the forest, Guinean and Sudano-Guinean zones. Protected areas representing each of those zones were visited as well as their surrounding areas, following the southwest and northeast ecological diagonal in Côte d’Ivoire:

- Taï National Park (TNP) and its surrounding areas: UNESCO World Heritage Site and Biosphere Reserve located in southwestern Côte d’Ivoire (5°08ʹ–6°24ʹN and 6°47ʹ–7°25ʹW). This park of 4540 km^2^ is one of the largest, relatively intact and well-conserved, upper evergreen rainforest remaining in West Africa (Eldin 1971). Annual rainfall ranges from a mean of 1700 mm in the north to 2200 mm in the southwest, falling from March/April to July, with a shorter wet season from September to October.
- Marahoué National Park (MNP) and its surrounding areas: Endangered National park located in west centre (7°05ʹ49ʹʹN–6°01ʹ32ʹʹO) 1010 km^2^. The vegetation is forest-savannah mosaic (Guinean savannah) with a mean annual precipitation varying between 1100 and 1800 mm. Two dry periods can be distinguished, stretching from November to February and from July to August. This park is covered with forest (2/3) and savannah. Four main habitat types are divided: open canopy forest, gallery forests, forest-savannah edge and savannahs (savannah woodland, tree savannahs and shrubby savannahs).
- Lamto Reserve (LR) and its surrounding areas: This natural reserve of 2500 ha is located in the transition zone between the semi-deciduous forest and the Guinean savannah (6°13″ N and 5°02″ W) in the so called “V”-Baoulé (central Côte d’Ivoire). The landscape of the Lamto Reserve is a forest-savannah mosaic (Menaut, 1971; Abaddie et al. 2006). The mean annual precipitation is about 1200 mm.
- Comoé National Park (CNP) and its surrounding areas: UNESCO World Heritage site and Biosphere Reserve, this park of 11,500 km^2^ is located northeastern (8°30ʹ–9°40ʹ N and 3°10ʹ–4°20ʹ W). The mean annual precipitation is around 1150 mm (Eldin 1971) with a mean annual temperature of 27°C. It is a semi-natural mosaic of forest-savannah (Sudano-Guinean savannah) with many habitats ranging from forests to savannahs, including all types of savannah (84%), bowal (4.9%), gallery forest (2.3%), and dry and humid forest islands (Poilecot et al. 1991; Hennenberg et al. 2005).

The Ivorian population is characterised by its great ethnic diversity, with various cultural and traditional considerations on one hand and diverse eating habits on the other hand. More than 60 ethnic groups belonging to four ethnolinguistic groups (Akan, Krou, Mandé and Voltaic) can be distinguished. However, the presence of some foreign population from border countries such as Burkina Faso, Ghana, Mali, Togo, Guinea and other West African countries, especially Nigeria, should be considered as well.

### Ethnomycological studies

The ethnomycological knowledge of rural populations of the major phytogeographical zones of Côte d’Ivoire was obtained using a structured questionnaire and casual conversations. The localities in which the ethnomycological studies were conducted were selected according to virtual transects taking into account all types of vegetation and climate of Côte d’Ivoire. During the surveys, a total of 1170 people from 39 localities and belonging to 20 ethnic groups were interviewed. The questionnaires and casual conservations were structured around six main points:

- the availability of *Termitomyces* fruiting bodies in the visited localities;
- indigenous knowledge and especially the uses of *Termitomyces* by local populations;
- diversity and abundance of *Termitomyces*;
- market value of *Termitomyces* fruit bodies and the identification of actors of a potential seasonal trade of these fruit bodies;
- periods and the fructification sites of *Termitomyces* fruit bodies;
- analysis of *Termitomyces*’s fruit bodies availability in the current context of climatic variability and land use types.

Based on these obtained indigenous knowledge on *Termitomyces*, the sampling design and efforts of fruiting bodies in natural habitats were properly refined and managed. Indeed, the fructification areas of *Termitomyces* were then marked out in Côte d’Ivoire with an estimate of the expected diversity in each of the visited phytogeographical zone. Furthermore, an overall view of the respective fructification periods of *Termitomyces* species reported by rural people within localities and regions was also obtained.

### *Diversity, phenology and distribution of* Termitomyces *and their respective host termites*

From 2011 to 2016, five field surveys were carried out in each of the major phytogeographical zone of the country. Sampling was done during the long and short rainy seasons as revealed by the ethno-mycological surveys and suggested by Koné et al. (2011, 2013). During each fruit bodies’ collecting season, *Termitomyces* habitats were revealed by ethonmycological surveys within each phytogeographical zone, and the works of Koné et al. (2011, 2013) as well.

Both termites (soldiers and workers) and fruit bodies of the symbiotic fungi were collected by breaking down each epigeal or subterranean mound until the fungus comb is exposed (Levieux 1967; Josens 1972; Konate 1998; Koné et al. 2011).

The descriptions of macroscopic features of the collected *Termitomyces* fruiting bodies were made on the fresh samples *in situ*, in order to record evanescent or changing characters when drying. These descriptions concerned the size and colour of fruit bodies, the presence or absence of perforatorium on the fruit body, the presence or absence of ring on the stipe, the colour of the stipe, the length of the stipe, the length of the pseudorhiza and the odour of the fruit body. Moreover, good photographs of specimens were made *in situ* using a high-precision camera.

Once at base camp, one spore print of each collected morphospecies was obtained by massively collecting spores on a blank sheet of paper. All specimens were then dried, labelled and packaged for laboratory analysis. The harvest date of each *Termitomyces* species was noted. Moist and dry collections of the fruiting bodies of each *Termitomyces* species were prepared and then kept in the laboratory.

### *Identification of* Termitomyces *fruiting bodies in the laboratory*

The identification of *Termitomyces* fruit bodies was done using the keys of Heim (1977) on termitophilic fungi from Africa and Southern Asia, the illustrations by Buyck (1994) of western Burundi’s edible mushrooms, the contribution of Mossebo et al. (2002) on the genus *Termitomyces* in Cameroon, the description of edible mushrooms from Benin (De Kesel et al. 2002) and the taxonomy and identification the fungi of central Africa, according to Eyi-Ndong et al. (2011).

Microscopic descriptions were made from exsiccata (dried specimens). Microscopic features such as basidia, cystidia (cheilocystidia and pleurocystidia) and gill trama were observed in Congo Red solution and spores in the Melzer’s reagent. All these observations were made using a Leica BMS photonic microscope equipped with a micrometer and a drawing tube.

### Host termite identification

The collected host termites (soldier caste) were identified to the species level under a low-power stereo microscope with a reticle (Nikon MZ6). Identification keys of African termites by Bouillon and Mathot (1965), Bouillon and Mathot (1966), 1971), Webb (1961), illustrations by Josens (1972) of Lamto savannahs termites, the different descriptions made by Grassé (1986) and the identification key of *Odontotermes* of the Lamto reserve by Konaté (1998) were used. Species with morphometric ambiguities were identified to the genus level.

## Results

### *Knowledge and uses of* Termitomyces *by populations in Côte d’Ivoire*

Ethnomycological knowledge of 1170 people from 39 localities and belonging to 20 ethnic groups was obtained. All the interviewees showed a solid knowledge of edible mushrooms in general and *Termitomyces* in particular. In northern Côte d’Ivoire, relatively little knowledge of mushrooms belonging to this genus was noted. However, those who knew these mushrooms had a good knowledge of the diversity, phenology and habitats of their respective phytogeographical zone. All the *Termitomyces* species reported by the populations interviewed were edible, with level of preference varying from one species to another. *Termitomyces letestui* (Pat.) R. Heim and *Termitomyces schimperi* (Pat.) R. Heim were identified as the most valued species. Populations of the Guinean zone noted that *Termitomyces fuliginosus* R. Heim required very good and long cooking so as to avoid indigestion to consumers. Finally, *T. medius* R. Heim & Grassé is generally considered by populations as a “dead wood mushroom”, i.e. saprotrophic species.

### *Diversity, phenology and distribution of* Termitomyces *in Côte d’Ivoire*

Fruit bodies of 16 *Termitomyces* species were collected; namely *T. letestui, T. medius, T. microcarpus* (Berk. & Broome) R. Heim, *T. striatus* (Beeli) R. Heim, *T. eurhizus* (Berk.) R. Heim, *T. fuliginosus* R. Heim, *T. schimperi, T. aff. aurantiacus* (R. Heim) R. Heim, *Termitomyces aff. clypeatus* R. Heim, *Termitomyces* sp1, *Termitomyces* sp2, *Termitomyces* sp3, *Termitomyces* sp4, *Termitomyces* sp5, *Termitomyces* sp6 and *Termitomyces* sp7. Nine new species of *Termitomyces* to Côte d’Ivoire and two potentially new species to science (*Termitomyces* sp1 and *Termitomyces* sp3) were recorded during this study.

The collected *Termitomyces* species were found associated with 15 species of host termites belonging to 5 genera (*Acanthotermes, Ancistrotermes, Macrotermes, Odontotermes* and *Pseudacanthotermes*) (Table 1). *Acanthotermes acanthothorax* and *Macrotermes subhyalinus* were respectively found in association with only one symbiotic *Termitomyces* species. The genera *Ancistrotermes* and *Pseudacanthotermes* were respectively found in association with two and three symbiotic species each. The highest number of symbiotic *Termitomyces* species (i.e. five species) was found in association with termites of the genus *Odontotermes. Termitomyces* species in association with this termite genus were found in the forest-savannah mosaic phytogeographical zone (LR, MNP and southwestern CNP). Two *Termitomyces* species were collected without their respective host termites (*Termitomyces* sp4 and *Termitomyces* sp6).

**Table 1.** Diversity and distribution of *Termitomyces* species and their respective host termite in Côte d’Ivoire.

		Distribution in Côte d’Ivoire			
		Forest zone (TNP)	Guinean zone		Sudano-Guinean zone (CNP)
*Termitomyces* species	Host termites		MNP	LR	
*Termitomyces letestui* (Pat.) R. Heim	*Pseudacanthotermes militaris*		X	X	
*Termitomyces medius* R. Heim & Grassé	*Ancistrotermes cavithorax/A. guineensis*	X	X	X	X
*Termitomyces fuliginosus* R. Heim	*Odontotermes* sp2		X	X	X
***Termitomyces schimperi*** (Pat.) R. Heim	*Macrotermes subhyalinus*	**X**			
*Termitomyces microcarpus (*Berk. & Broom) Heim	*Odontotermes* sp1	X			**X**
***Termitomyces eurhizus*** (Berk.) R. Heim	*Acanthotermes acanthothorax*	**X**			
***Termitomyces* cf. *clypeatus*** R. Heim	*Ancistrotermes guineensis*				**X**
***Termitomyces aurantiacus* (**R. Heim) R. Heim	*Odontotermes* sp5				**X**
***Termitomyces* cf. *striatus*** (Beeli) Heim	*Ancistrotermes cavithorax*		**X**		
***Termitomyces* sp1**	*Pseudacanthotermes minor*	**X**			
***Termitomyces* sp2**	*Odontotermes* sp4		**X**		
***Termitomyces* sp3**	*Pseudacanthotermes spiniger*		**X**		
***Termitomyces* sp4**	Undetermined		**X**		
***Termitomyces* sp5**	*Ancistrotermes* sp.		**X**		
***Termitomyces* sp6**	Undetermined		**X**		
***Termitomyces* sp7**	*Odontotermes* sp3		**X**		
Total		5	10	3	5

Abbreviations: CNP = Comoe National Park; LR = Lamto Reserve; MNP = Marahoue National Park; TNP = Taï National Park; Bold entries correspond to fungal species exclusively found in a single phytogeographical zone.

The fructifications of *Termitomyces* species were observed during the rainy seasons with the exception of *T. eurhizus* whose fruit bodies were observed at the beginning of the long dry season (Table 2). However, each species had a specific fructification period even if the fructification periods of some species overlapped. Furthermore, the span times of the fructification periods also differ from a species to another. Indeed, the longest fructification periods were observed with *T. letestui, T. medius* and *T. fuliginosus* during the long rainy season. On the contrary, the fruit bodies of *T. schimperi* and *T. eurhizus* were observed during the short rainy season.

**Table 2.** Mean occurrence periods of *Termitomyces* species in Côte d’Ivoire.

Species	Months											
	J	F	M	A	M	J	J	A	S	O	N	D
*Termitomyces letestui* (Pat.) R. Heim												
*Termitomyces medius* R. Heim & Grassé												
*Termitomyces* cf. *fuliginosus* R. Heim												
*Termitomyces schimperi* (Pat.) R. Heim												
*Termitomyces microcarpus (*Berk. & Broom) Heim												
*Termitomyces* cf. *eurhizus* (Berk.) R. Heim												
*Termitomyces* cf. *clypeatus* R. Heim												
*Termitomyces aurantiacus* (R. Heim) R. Heim												
*Termitomyces* cf. *striatus* (Beeli) Heim												
*Termitomyces* sp1												
*Termitomyces* sp2												
*Termitomyces* sp3												
*Termitomyces* sp4												
*Termitomyces* sp5												
*Termitomyces* sp6												
*Termitomyces* sp7												
Phenological groups	ES (1)			MSS (14)							LS (1)	

J, F, M, A, M, J, J, A, S, O, N, D represent the months from January to December. ES = early species; MSS = mid-season species; LS = late species.

The identification of the respective fructification periods of the collected species enabled to classify them in three major groups namely the early species (*T. letestui*) which fruit bodies are observed with the first rains of the year, the intermediate species (*T. medius*) which fructification occurs during the rainy season and the late species (*T. schimperi* and *T. fuliginosus*) which fruit bodies occur at the end of the rainy season (Table 2).

In total, 5 species were recorded in the south of the country (forest zone), 10 species in the centre (forest-savannah mosaic zone) and five species in the Sudano-Guinean zone in the northeast (Table 2). *Termitomyces medius* was found in all phytogeographical zones. Three species (*T. medius, T. microcarpus* and *T. fuliginosus*) were found in at least two of the visited phytogeographical zones. The highest diversity of *Termitomyces* was observed in the Guinean savannah zone in the Marahoué National Park and the Lamto Reserve (Table 2). Eight species were exclusively encountered in this phytogeographical zone (*T. letestui, T. striatus* and *Termitomyces* sp2, *Termitomyces* sp3, *Termitomyces* sp4, *Termitomyces* sp5, *Termitomyces* sp.6 and *Termitomyces* sp7). The forest zone (TNP) had three exclusive species (*T. schimperi, T. eurhizus* and *Termitomyces* sp1) while only two species (*T. aurantiacus* and *T. clypeatus*) were exclusively found in the Sudano-Guinean zone (CNP).

## Discussion

### *Socio-economic importance of* Termitomyces*’ fruit bodies*

This work has shown that the Ivorian populations have a great knowledge of the fungal genus *Termitomyces* regardless of the visited phytogeographical zone. This unusual ethnomycological knowledge helped to properly refine and design the sampling protocol of the study by reducing sampling efforts through the identification of fructification habitats and periods of *Termitomyces* species within the visited phytogeographical zones. *Termitomyces letestui* and *T. schimperi* are the most valued species. Indeed, fruit bodies of both species are highly appreciated and very much sought after food source in the extend savannah-forest boundaries, from the Eastern to the Western part of the country. For local people fruit bodies also appear at a crucial moment of the rainy season, i.e. when food stocks are dwindling and when the new crops are not at full maturity. The ignorance of certain species by populations could be explained by the scarcity of their respective fructifications. Indeed, *T. eurhizus* for example was collected only once throughout this study.

### *Diversity and distribution of* Termitomyces *in Côte d’Ivoire*

This study revealed that Côte d’Ivoire has the greatest diversity of *Termitomyces* in West Africa, with 16 collected species. Indeed, eight species were recorded in Benin (Yorou et al. 2002), six species in Togo (Kamou et al. 2015). However, the higher sampling effort in Côte d’Ivoire when compared to the other West African countries needs to be kept in mind. Moreover, Buyck (1994) mentions 4 species for Western Burundi while Mossebo (2002) estimates this species richness to 14 species in Cameroon and 6 species for Zambia (Pegler and Pearce 1908). Morris (1986) recorded eight species in Malawi and seven species in South Africa.

The centre of origin hypothesis introduced by Darwin as “centres of creation” (Darwin 1859) proposed that species originate in a centre and disperse from it to the periphery. The centre of origin as the centre of diversity theory was first developed by Vavilov (1927) for the origin of crops. Several studies across taxa have shown that the centres of origin are often centres of the genetic diversity (e.g. Banke and McDonald 2005; Stukenbrock et al. 2007; for plant pathogens; Moran et al. 2003; for reef fishes). The centre of origin of termite fungiculture, under this hypothesis, would then be the centre of higher diversity of both fungus-growing termites and *Termitomyces*. Côte d’Ivoire could then be considered as this centre of origin, as suggested by Koné (2013). Indeed, this author used *Termitomyces* nodules as well as fruit bodies and claimed an expected global diversity of 20 species of *Termitomyces* for this country.

The diversity of *Termitomyces* from Côte d’Ivoire differs from one phytogeographical zone to another, showing that these mushrooms have very specific fructification habitats as suggested by Koné et al. (2011). Indeed, fruit bodies of these fungi occur in wooded habitats species, specifically either during the long or the short rainy season.

The greatest diversity of *Termitomyces* fruiting bodies was recorded in the forest-savannah mosaic phytogeographical zone. *T. eurhizus, T. schimperi* and *Termitomyces* sp1 were exclusively found in the forest zone; whereas *T. aff. aurantiacus* and *T. clypeatus* were found in the Sudano-Guinean zone. The extend savannah-forest boundaries, from the Eastern to the Western part of the country, seems to be their main occurrence zone in Côte d’Ivoire.

Finally, *T. medius* was observed as common species to all phytogeographical zones. This could be explained by the ecology of its respective host termites (Aanen and Eggleton 2005; Koné 2013). Indeed, the local specific richness of termites is strongly influenced by the variability of environmental factors including altitude (Gathorne-Hardy et al. 2001), vegetation type (Jones, 2000), anthropogenic disturbances (De Souza and Brown 1994; Eggleton et al. 1995, 1997, 2002) and climate (Eggleton and Bignell 1995). Termites of the genus *Ancistrotermes* are endemic to savannahs in the afrotropical region (Eggleton 2000). They also are very resilient to natural disturbance and might actually benefit from some natural disturbances.

### *Phenology of* Termitomyces *species*

Each *Termitomyces* species was collected at a specific fructification period. *T. letestui* was observed with the first rains of February until the end April. It overlapped with the fructification period of the common species *T. medius*. Overlapping fructification periods of some species was observed. This overlap of fructification could be related to the amount of rain that has fallen. According to Koné et al. (2013), the triggering of *Termitomyces* fructification is caused by direct or indirect effects depending on the host termites food requirements. Moreover, a non-overlapping period of fructification of most species was observed. According to Koné et al. (2011), this lack of overlap could be considered as a choice of host termites allowing them easier and simpler recognition of the spores of their respective symbiotic mushroom.
